# Mechanism study of oleanolic acid derivative, K73-03, inducing cell apoptosis in hepatocellular carcinoma

**DOI:** 10.1186/s12935-023-03119-x

**Published:** 2024-01-07

**Authors:** Jiaqi Wang, Chuchu Ma, Zhongyuan Tang, Zhengwu Sun, Eskandar Qaed, Xinming Chi, Jun Wang, Yazeed Jamalat, Zhaohong Geng, Zeyao Tang, Qiying Yao

**Affiliations:** 1 Department of Physiology, Dalian Medical University, Dalian, China; 2 Department of Plastic and Reconstructive Surgery, The First Hospital of Jilin University, Changchun, Jilin 130000 China; 3 Department of Pharmacology, Dalian Medical University, Dalian, China; 4 Department of Orthodontics, College of Stomatology, Jilin University, Changchun, Jilin 130033 P.R. China; 5 Histology and Embryology Department, Dalian Medical University, Dalian, China; 6 Pathophysiology Department, Dalian Medical University, Dalian, China; 7 Department of Cardiology, 2th Affiliated Hospital of Dalian Medical University, Zhongshan Road No. 467, Dalian, 116000 China

**Keywords:** Oleanolic acid derivative, K73-03, Hepatocellular carcinoma, Apoptosis, JAK2/STAT3, NF-κB/P65

## Abstract

Oleanolic acid (3β-hydroxyolean-12-en-28-oic acid, OA) is a kind of pentacyclic triterpene, which widely distributes in nature. OA possesses a powerful anti-cancer effect; however, its low solubility limits its bioavailability and application. In this study, a new OA derivative, K73-03, was used to determine its effect on liver cancer cells and detailed molecular mechanisms. Here, we show that K73-03 may lead to the disorder of mitochondria in HepG2 cells, leading to excessive ROS production and apoptosis in cells. Meanwhile, K73-03 could induce cell apoptosis by inhibiting JAK2/STAT3 pathway and NF-κB/P65 pathway. Collectively, this study may provide a preliminary basis for further cancer treatment of hepatocellular carcinoma.

**Supplementary Information:**

The online version contains supplementary material available at 10.1186/s12935-023-03119-x.

## Introduction

Liver cancer is one of the most common cancers in the world. Its incidence rate is very high in Asia. Moreover, in recent years, the incidence rate of liver cancer is increasing year by year, rising to the fifth most common cancer. The mortality rate is only after lung cancer and gastric cancer. The estimated incidence rate of liver cancer is about 500 thousand to 1 million per year, causing 600 thousand of deaths worldwide annually [1, 2]. For patients with early-stage liver cancer, local destructive treatment and liver surgery are the main effective treatment methods. However, most patients are diagnosed with advanced liver cancer and do not have the opportunity for surgery. This is mainly because the early diagnosis of liver cancer is very difficult, leading to the majority of patients with liver cancer being diagnosed as an advanced stage. At present, the efficacy of traditional systemic chemotherapy is very low, and the side effects are great [3, 4]. Moreover, the liver cancer recurrence rate following 5 recurrence-free years after liver cancer treatment is high [5]. Therefore, the development of new anti-liver cancer drugs with reliable effects is of great significance for the adjuvant treatment of early liver cancer surgery and the conservative treatment of advanced liver cancer.

Traditional Chinese medicine (TCM) has the characteristics of low toxicity and side effects, which is often used in the treatment of various types of cancer in recent years. Many pentacyclic triterpenoids, such as oleanolic acid (OA), have been widely studied for their good biological activity. OA in nature often exists in the form of free or glycoside type, which can be extracted from thousands of medicinal and edible plants [6]. The most important and widely used pharmacological action of OA is its hepatoprotective effect. OA can not only effectively avoid acute chemical-induced liver injury, but also protect the liver from cirrhosis and fibrosis caused by chronic liver disease. In addition to liver protection, in recent years, researchers have found that OA has anti-inflammatory, antioxidant, hypoglycemic and anti-tumor effects. Some studies have confirmed that OA possesses an anti-tumor effect on some human tumor cell lines, including human lung cancer A549, NCI-H460 cell lines, human astrocytoma 1321N1 cell lines and a variety of human liver cancer cell lines [7]. It has proved that OA can induce cell differentiation and apoptosis, and inhibit cancer angiogenesis and cancer cell invasion and metastasis [8]. However, the bioavailability of OA in vivo still is limited because of its low solubility. Therefore, though synthesizing some OA derivatives, such as CDDO, CDDOEA, CDDOIm, CDDOMe, etc., the low solubility of OA has been improved to some extent [9]. For example, CDDO-Me has shown a promising anticancer effect in a phase I trial. But, some transient and self-limiting side effects still exist, such as an increase in adverse cardiovascular events [10]. Hence, developing more new OA derivatives with good anti-hepatic cancer effects is of great significance. In our laboratory, we previously synthesized several novel OA derivatives, called SZC014, SZC015, and SZC017, and evaluated their antitumor effects against different cancer cells [11–18]. However, the effects of K73-03, a new OA derivative, on liver cancer especially for in vitro cancer suppression activity, have not been investigated.

Programmed cell death is defined as regulatory cell death mediated by an intracellular program. Apoptosis and autophagy in cells are two main mechanisms for inducing programmed cell death [19]. However, in cancer, they are always out of balance [20]. Apoptosis is a type I programmed cell death form performed by activating caspases, which is a specific enzyme involved in the signal cascade, eventually leading to the rapid elimination of organelles and other cell structures [21, 22].

Janus kinase 2/signal transducer and activator of transcription 3 (JAK2/STAT3) signaling pathway plays an important role in a variety of cancers, including prostate cancer, hematological malignancies, and liver cancer [23]. In liver cancer, JAK / STAT signaling pathway is upregulated in tumor tissues compared to normal tissues [24]. It has been found that methylation-induced silence of SOCS1 and SOCS3 (a negative regulator of JAK / STAT signal transduction) are observed in most liver cancer cells, resulting in the activation of JAK / STAT signal transduction, thus increasing the growth of liver cancer cells and resistance to apoptosis [25].

In this study, HL-7702(L02), SMMC-7721 and HepG2 cells were used to explore and elucidate the anti-tumor mechanism of a new OA derivative K73-03 in liver cancer. The objective is to explore whether K73-03 could induce apoptosis or autophagy in liver cancer cells and clarify its relationship with JAK2 / STAT3 signaling pathway or other possible mechanisms.

## Materials and methods

### Chemicals and materials

K73-03 was synthèsed by Professor Shi sheng Wang from Dalian University of Technology. OA (2.0 mg) and K73-03(2.0 mg) were dissolved by DMSO. They were stored at − 20℃. Dulbecco’s modified Eagle medium (DMEM) was purchased from Gibco (Grand Island, NY, USA). Uncoupler FCCP, rotenone and antimycin A were purchased from Sigma (St Louis, MO, USA). The AnnexinV-FITC Apoptosis Detection Kit, JC-1 fluorescent molecular probe and DCFH-DA were obtained from the Nanjing Jiancheng Bioengineering Institute (Haimen, Jiangsu, China). The BCA protein concentration assay kit, IP lysate and Hypersensitive ECL chemical kit were obtained from the Beyotime Institute of Biotechnology (Haimen, Jiangsu, China). The primary antibodies against caspase-9, caspase-3, Bcl-2, Bax, LC3B, JAK2, p-JAK2, p65, p-p65 were pased from Proteintech (Chicago, IL, USA). The antibodies Beclin1, STAT3, p-STAT3, COX-2 were obtained from the Bioworld (Minnesota, St Paul, USA).

### Cell culture

The human normal liver cell line L02, and the human HCC cell line SMMC-7721 and HepG2 were kindly provided by Professor Wang Shi Sheng at Dalian University of Technology. All cell lines were cultured in DMEM supplement with 10% fetal bovine serum, 100 units/mL penicillin and 100 µg/mL streptomycin. All cells cultures were maintained at 37 ℃ in a humidified atmosphere containing 5% CO_2_.

### MTT assay

Cell viability was measured using an MTT assay. L02, SMMC-7721 and HepG2 cells were seeded at 7 × 10^3^ cells/well in 96-well plates (2.19 × 10^4^ cells/cm^2^) for 24 h. And then, different concentrations of K73-03 (0, 2, 4, 8 µM) were added into plates. After 24 h incubation, the growth of cells was measured. And then cells were treated with 15µL MTT stock solution (5 mg/mL) added to the well. Additional 4 h later, 100µL SDS-isobutanol-HCl solution was added and the plates were further incubated at 37℃ overnight. Absorbance was measured at 570 nm by a microplate reader. Additionally, cell morphology was imaged using a readout microscope.

### In vitro migration assay

Cell migration was detected by scratch assay. HepG2 cells were seeded at 5 × 10^5^ cells/well in 6-well plates (5.26 × 10^4^ cells/cm^2^), and then wounded with 100 µL steriled pipette tips after 6 h of serum starvation. After three PBS washes, cells were treated with indicated doses of K73-03. Then, treated cells were photographed using the light microscope for 0 h and then were contained with fresh medium in a 37 ℃, 5% CO_2_ incubator. After 48 h, the cells were washed with PBS three times and then were photographed again using the light microscope.

### Colony formation assay

HepG2 cells were seeded into 6-well plates at 0.8 × 10^3^ cells/well (84.2 cells/cm^2^). After 24 h, the cells were exposed to various concentrations of K73-03 (0, 2, 4, 8µM). After 24 h, the cells were washed with PBS three times and then contained with fresh medium in a 37 ℃, 5% CO_2_ incubator. Until 14 days, the cells were immobilized by 4% paraformaldehyde and then stained with 0.1% crystal violet.

### Transmission electron microscope

HepG2 cells were cultured in 6-well plates for 24 h. And then the cells were treated with different concentrations. The samples were post-fixed, dehydrated, embedded, sectioned, and stained. Finally, the electron micrographs were captured using a Transmission Electron Microscope (TEM).

### DAPI staining

HepG2 cells were cultured in 6-well plates for 24 h and treated with various concentrations of K73-03 (0, 2, 4, 8µM) for 24 h. The cells were washed with PBS three times and fixed with paraformaldehyde 15 min. Samples were permeabilized with 0.4% TritonX-100 for 10 min and washed with PBS three times, which was sealed with 2% bovine serum albumin (BSA) in PBS for 1 h at 37℃. Finally, the samples were stained with DAPI (1 µg/mL) for 15 min, and then washed with PBS for 3 times. The changes of blue fluorescence intensity in nucleus were observed and photographed under an inverted fluorescence microscope.

### Apoptosis assay (annexin V-FITC/PI double staining)

The effect of K73-03 on HepG2 cells was detected using Annexin V-FITC Apoptosis Detection Kit. HepG2 cells were cultured in 6-well plates for 24 h and treated with various concentrations of K73-03 (0, 2, 4, 8µM) for 24 h. And then the cells were stained with Annexin V-FITC and propidium iodide (PI) for 15 min in the dark at 37℃, the samples were subsequently analyzed using Calibur flow cytometry.

### JC-1 fluorescent molecular probe

JC-1 is a type of fluorescence probe for detecting mitochondrial membrane potential (∆Ψm). HepG2 cells were cultured in 6-well plates for 24 h and treated with various concentrations of K73-03 (0, 2, 4, 8µM) for 24 h. The cells were washed with PBS three times and then added with JC-1(1mL). After 15 min, the samples were observed by an inverted fluorescence microscope.

### Intracellular reactive oxygen species (ROS) level detection

The level of ROS production was measured by intracellular ROS level detection. Briefly, HepG2 cells were cultured in 6-well plates for 24 h and treated with various concentrations of K73-03 for 24 h. And then the cells were harvested and cultured with DCFH-DA at 37℃ for 20 min in the dark. After washing with PBS three times, the samples were monitored by FACS flow cytometry.

### Detection of cell mitochondrial function

Oxygen concentration and oxygen flux or oxygen consumption rate (OCR) were measured using oxygraph-2k mitochondrial function test system. The cells in the normal control group and the drug group with a density of 2 × 10^6^ / mL were added into two glass chambers, and the experiment was carried out under the condition of continuous stirring at 37 ℃ and 750 rpm. After the basal respiratory rate was recorded, inhibitors of different mitochondrial respiratory complexes were added to the cells in the following order: (1) 2 µ g / mL oligomycin (acting on ATP synthase and measuring mitochondrial oxygen consumption unrelated to ATP synthesis); (2) gradually increasing the amount of 1 µ M into carbonyl cyano- p-trifluoromethoxyhydrazone (FCCP) to evaluate Maximum respiratory rate (MRC) 5 µ M, respectively, to determine the capacity of non-respiratory complex (5 µ M).

### Western blot analyses

HepG2 cells were cultured in 6-well plates for 24 h and treated with various concentrations of K73-03 for 24 h. The cytoplasmic and nuclear protein samples were measured by cell lysis kits. Protein was denatured by dissolving in 5× sample buffer and boiled for 5 min at 100 ℃. And then samples were loaded onto the SDS-PAGE, separated by gel electrophoresis and transferred to PVDF membranes. After blocking with 5% skimmed milk in Tris Buffered Saline (TBS) containing 0.05% Tween 20 (TTBS) for 2 h, the membranes were incubated overnight at 4 ℃ with primary antibody (1:1000 dilution). Next, the blots were washed by TBST three times, then the membranes were cultured in horseradish peroxidase-conjugated secondary antibody (1:1000 dilution) at 4 ℃. After 2 h, the membranes were washed three times with TBST. In the end, the membranes were detected by using an enhanced chemiluminescence method and photographed by BioSpectrum Gel Imaging System. The data were adjusted by β-actin as an internal control.

### Statistical analyses

All the data were analyzed using Graph Pad Prism 6 (Graph Pad Software, Inc, San Diego, CA) and expressed as mean ± SD from at least three independent experiments. A one-way ANOVA test was utilized to analyze statistical differences between groups. *P* value < 0.05 was regarded as statistically significant.

## Results

### K73-03 inhibited the cell viability of L02, HepG2 and SMMC-7721

**Fig. 1:**
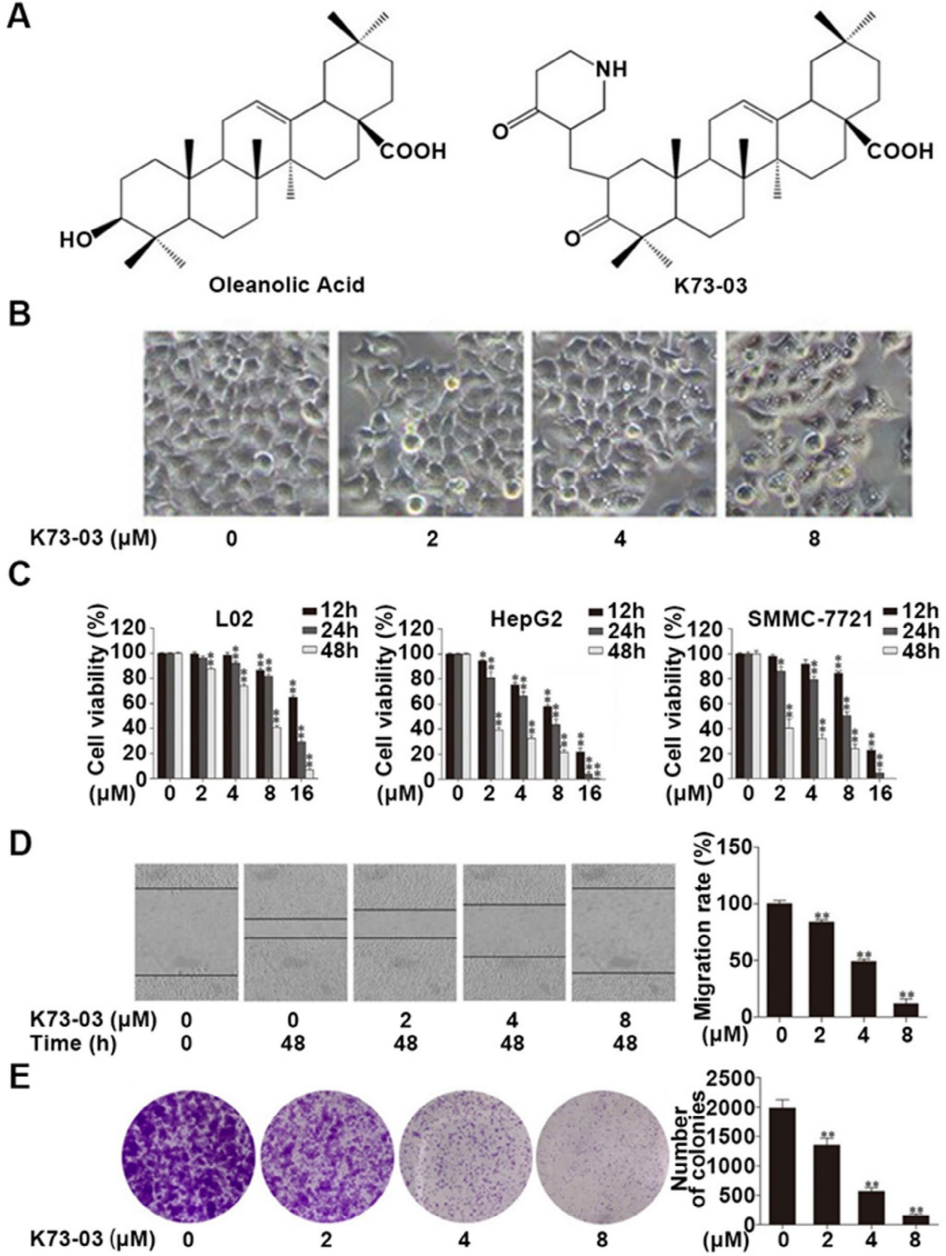
The inhibitory effect of K73-03 on L02, HepG2 and SMMC-7721 cells. (**A**) The chemical structure of OA and K73-03. (**B**) The effect of K73-03 on morphological changes and cell viability of cell lines. (**C**) The effect of K73-03 concentration and time on cell survival rate after treatment with K73-03 (0, 2, 4, 8, 16 µM) for 12, 24, 48 h via MTT assay respectively. (**D**) K73-03-induced HepG2 Cell migration was analyzed by a wound-healing assay. (**E**) K73-03-induced colony formation was also analyzed, and the colony formation rate was calculated. The data were presented as the mean ± SD of three independent experiments. (*p < 0.05, **p < 0.01, significantly different from control group)

The chemical structures of OA and OA derivative, K73-03, are represented in Fig. 1A. To determine the specificity of K73-03 on HCC, we examined the effect of K73-03 on morphological changes and cell viability of L02, HepG2 and SMMC-7721 cell lines. The effect of apoptotic morphological changes on HepG2 after K73-03 treatment was evaluated by microscopic observation as shown in Fig. 1B, including cell shrinkage and fragmentation, accompanied by occurring white fragments with different sizes and irregular shapes beside cells, and so on. Meanwhile, via MTT assay, the data showed K73-03 could largely decrease the cell viability of HepG2 and SMMC-7721 cell lines in a time and dose-dependent manner. The half maximal inhibitory concentration value (IC_50_) values of K73-03 for 24 h of HepG2 and SMMC-7721 cell lines were 5.79 µM and 7.23µM respectively. Moreover, the treatment of K73-03 caused a significant reduction in cell viability of HepG2 and SMMC-77,721 with the augment of concentrations and the extension of drug administration times. Compared to these two HCC cell lines, K73-03 had less toxicity to normal liver cell, L02 mammary epithelial cell line. Therefore, the data suggested that K73-03 could potentially affect cell viability and kill liver cancer cells, especially to HepG2 cells (Fig. 1C).

### K73-03 suppressed migration and colony formation of HCC cells

As shown in Fig. 1D, the effect of K73-03 on the migration of HepG2 cells was observed via wound healing assay. Based on the IC50 value of K73-03 on HepG2, four different concentrations of K73-03 (0, 2, 4, 8µM) were used for following experiments so as to take efficacy and safety into consideration. The data showed that K73-03 treatment could significantly inhibit the migration of HepG2 cells in a dose-dependent manner, compared to the non-treatment group. Meanwhile, the effect of K73-03 on the colony capacity of HepG2 cells was also measured using a colony cell survival assay. The result showed that K73-03 could significantly inhibit colony formation in a dose-dependent manner (Fig. 1E). These suggested that K73-03 could suppress the migration and colony formation of HepG2 cells.

### K73-03 induced apoptosis in the HCC cells

**Fig. 2:**
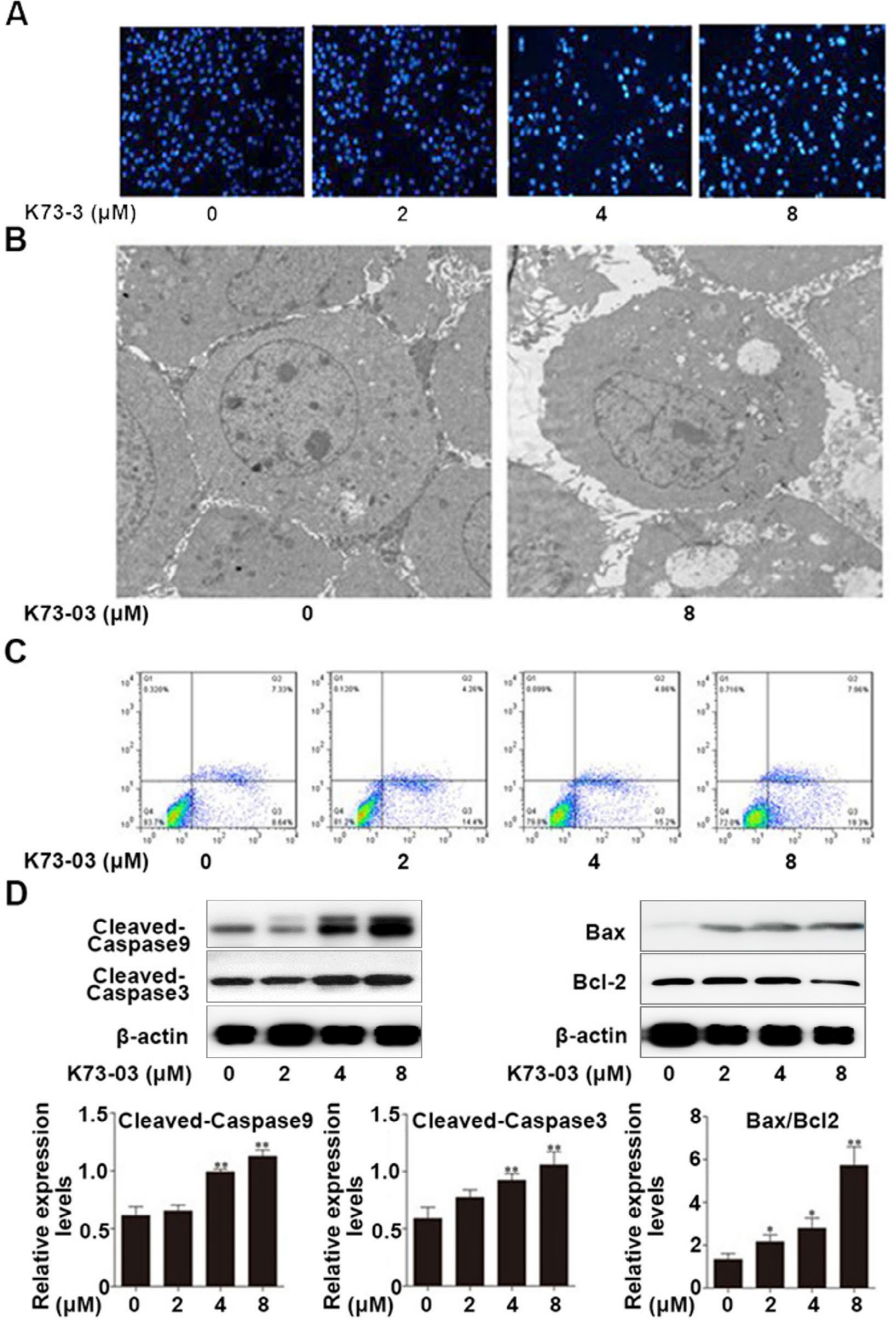
Induction of apoptosis by K73-03 in HepG2 cells. (**A**) Apoptotic morphological changes on HepG2 after K73-03 treatment (0, 2, 4 and 8 µM) using DAPI staining. (**B**) TEM observed the occurrence of apoptosis in HepG2 cells after treatment with 8 µM K73-03 for 24 h. (**C**) The evaluation on apoptotic rate using Annexin V-FITC and PI double staining analyzed by flow cytometry. (**D**) The expressions of cleaved-caspase-9, cleaved-caspase-3, Bax and Bcl-2 protein were determined by Western blotting. The data were presented as the mean ± SD of three independent experiments. (*p < 0.05, **p < 0.01, significantly different from control group)

The effect of apoptotic morphological changes on HepG2 after K73-03 treatment was evaluated by microscopic observation using DAPI staining. As shown in Fig. 2A, K73-03 induced apoptosis in HepG2 cells in a concentration-dependent manner, including cell shrinkage and fragmentation so on. The ultrastructure was observed by TEM. Figures showed that the cells in the non-treatment group were normal, while the cells in K73-03 treatment presented apoptotic morphology, becoming an abundant of vacuoles, nucleus chromatin condensation and marginalization, narrowing of nuclei and loss of microvilli (Fig. 2B).

To evaluate the apoptotic induction by K73-03 in the HepG2 cells, Annexin V-FITC and PI double staining were analyzed by flow cytometry. As shown in Fig. 2C, K73-03 induced the apoptosis of HepG2 cells in a concentration-dependent manner. Moreover, the K73-03 treatment significantly increased the early apoptotic rate from 8.64 to 19.3% and the total apoptotic rate from 15.97 to 27.26%. To further determine whether caspases were activated in K73-03-induced apoptosis, the levels of cleaved caspase-9 and cleaved caspase-3 of K73-03-treated HepG2 cells were evaluated by Western blot. As shown in Fig. 2D, K73-03 could significantly increase the expression of cleaved caspase-9 and cleaved caspase-3. Then, the relationships of K73-03-induced apoptosis with the expression of anti-apoptotic protein Bcl-2 and pro-apoptotic protein Bax were also confirmed, showing that K73-03 could significantly increase the Bax/Bcl-2 ratio. The results suggested that activation of caspases and an increase in the Bax/Bcl-2 ratio may be a possible mechanism of K73-03 induced apoptosis in HepG2 cells.

### K73-03 induced autophagy in HepG2 cells

**Fig. 3:**
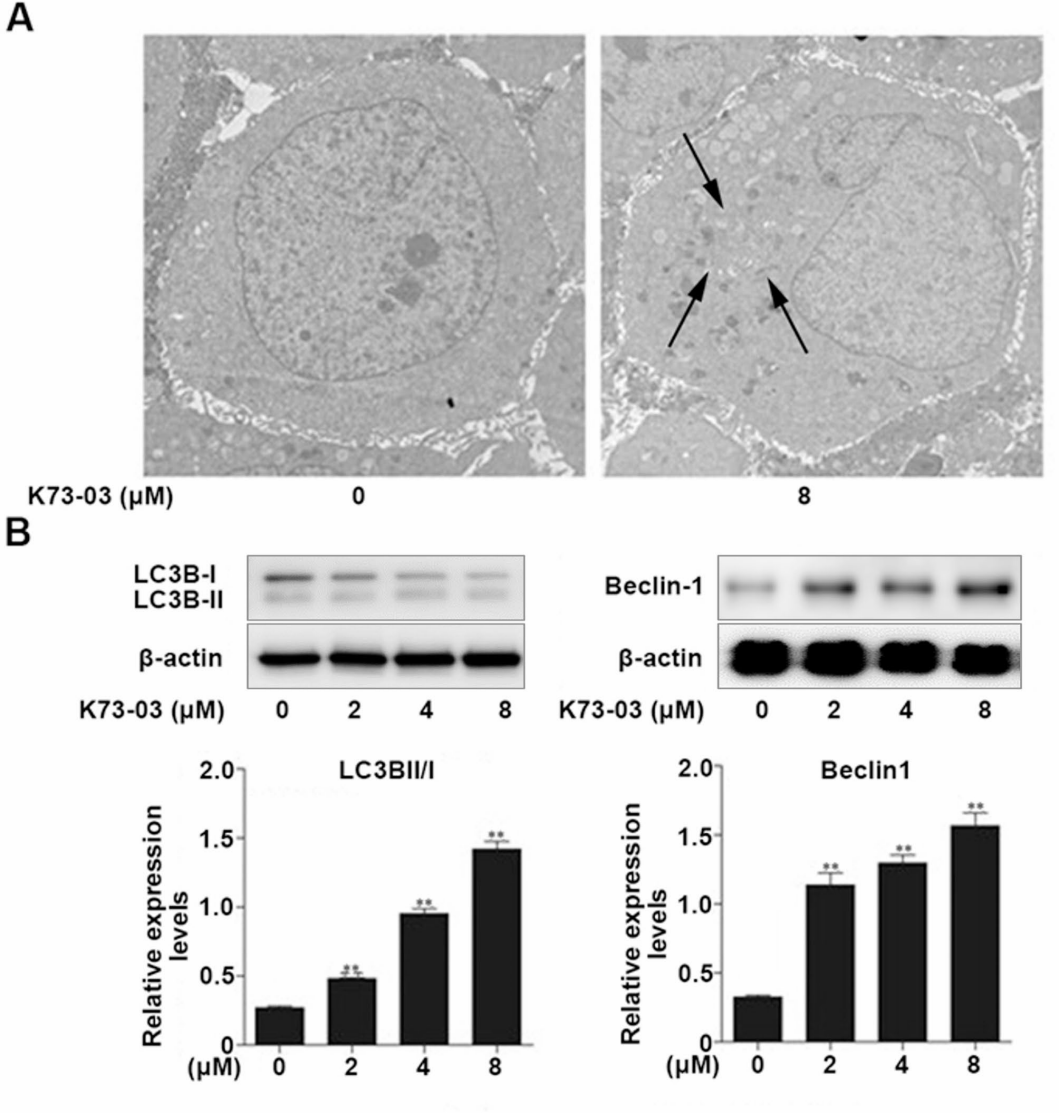
Induction of autophagy by K73-03 in HepG2 cells. (**A**) After treatment with 8 µM K73-03 for 24 h, the occurrence of autophagosome in HepG2 cells was observed by TEM. (**B**) The expressions of the autophagy protein LC3B and Beclin-1 in HepG2 cells were analyzed by Western blotting. The data were presented as the mean ± SD of three independent experiments. (*p < 0.05, **p < 0.01, significantly different from control group)

Autophagy is a highly conservative process to maintain cell homeostasis. The cytoplasmic contents can be isolated and transported to the lysosome through the membrane autophagosomes. TEM is the best standard method to assess autophagy. As shown in Fig. 3A, the ultrastructure in HepG2 cells could be observed after the treatment of K73-03. Compared with the normal group, autophagosomes appeared in HepG2 cells of the K73-03 treatment group, indicating that K73-03 could induce HepG2 autophagy. The autophagy was further confirmed using western blot. To determine whether autophagic-related proteins were attached to K73-03-induced autophagy, the levels of LC3B-II/LC3B-I and Beclin 1 in K73-03-treated HepG2 cells were evaluated by western blot. As shown in Fig. 3B, K73-03 could significantly increase the expression of the ratio level of LC3B-II/LC3B-I and Beclin 1.

### Effect of K73-03 on mitochondrial function of HepG2 cells

**Fig. 4:**
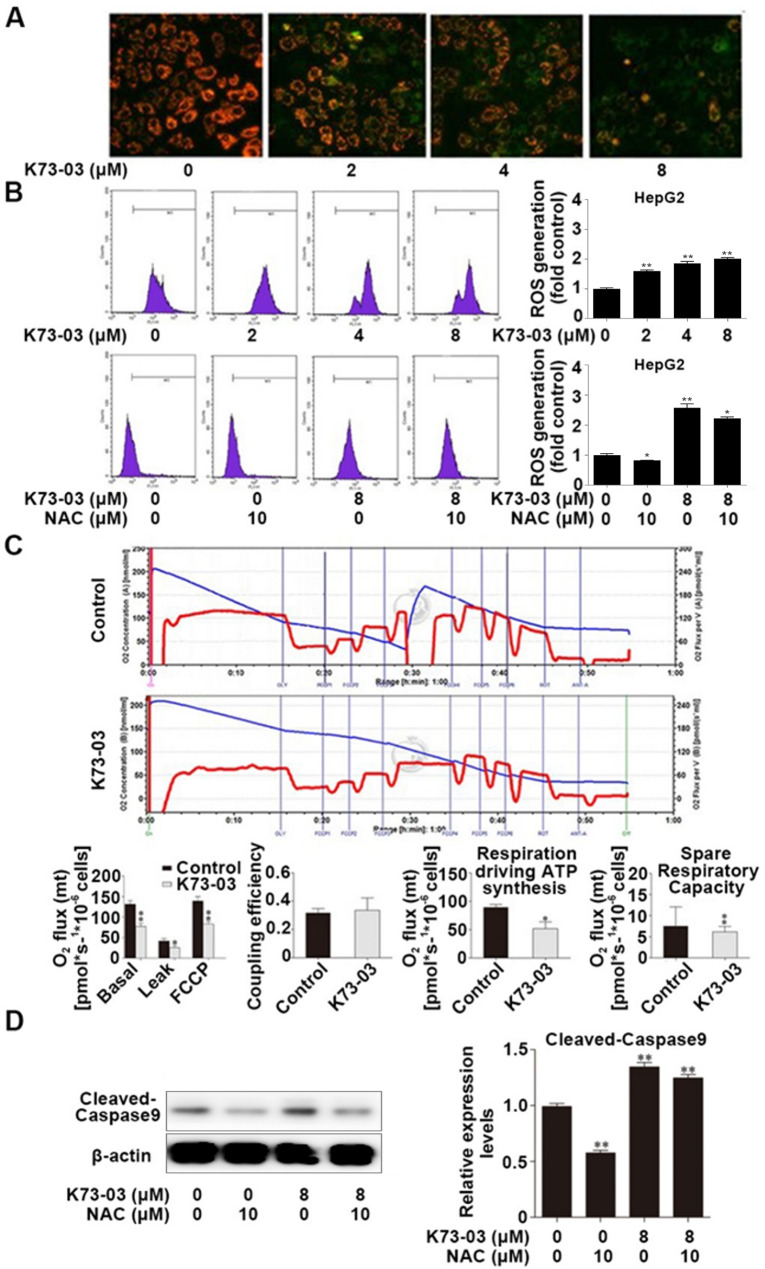
Effect of K73-03 on mitochondria of HepG2 cells. (**A**) Changes of HepG2 ∆Ψm induced by K73-03 were detected by JC-1. (**B**) Effect of excessive ROS generation in K73-03-treated HepG2 cells using flow cytometry. (**C**) Results of and FCCP/ MRC, coupling efficiency, respiration driving ATP synthesis/Oligomycin sensitive OCR and spare respiratory capacity in HepG2 cells. (**D**) After pretreatment with 10 µg/mL NAC for 1 h, the expression of cleaved- caspase-9 was analyzed by Western blotting. The data were presented as the mean ± SD of three independent experiments. (*p < 0.05, **p < 0.01, significantly different from control group)

Changes in HepG2 ∆Ψm induced by K73-03 were detected by JC-1. As shown in Fig. 4A, the fluorescence of JC-1 was gradually changed from orange to green with the increasing concentration of K73-03, suggesting that the ∆Ψm was gradually decreased with the increase of concentration of K73-03.

Mitochondrial failure can lead to a significant increase in ROS overproduction, and excessive ROS acts on the mitochondrial stress pathway, thus inducing cancer cell apoptosis. Results indicated that K73-03 could affect mitochondrial function and induce apoptosis of HepG2 cells through the mitochondrial pathway. After 24 h K73-03 treatment for HepG2, the formation of ROS was investigated by flow cytometry. K73-03 could increase ROS content in a dose-dependent manner (Fig. 4B). N-acetyl-L-cysteine (NAC) is an active oxygen scavenger. In this study, NAC was used as a positive control to further determine the effect of K73-03 on ROS production. As shown in Fig. 4B, the ROS level in HepG2 cells was decreased after NAC treatment, while the ROS level was increased significantly after pretreating NAC for 1 h and then adding K73-03.

Since K73-03 triggers intrinsic apoptosis, we aimed to identify key upstream events leading to this phenomenon. As shown in Fig. 4C, the baseline OCR of the control group was higher than that of the K73-03-treated group. Additionally, some other parameters derived from the assays were calculated. Oligomycin-resistant respiration rates are most attributed to proton leakage, whereas basal OCR is affected by ATP synthesis, proton leakage, and substrate oxidation. Then, K73-03 significantly reduced the oligo-OCR compared to controls, which could be a combination of proton leakage, mitochondrial electron transport chain (ETC) uncoupling, or due to membrane damage or at the expense of electrochemical gradients metabolite transport [26]. Differences in coupling efficiency were also observed, suggesting that ETC decoupling is not a major contributor to proton leakage. The results showed that oligomycin-sensitive OCR (respiration-driven ATP synthesis) was significantly reduced in K73-03-treated cells, indicating a decrease in complex V ATP synthesis. As further evidence of increased mitochondrial reserves, cancer cells had a higher reserve respiratory capacity than K73-03-treated cells, suggesting that K73-03 treatment was unable to increase energy production in the face of increased demands. Therefore, these results suggested that K73-03 may cause mitochondrial damage in HepG2 cells. Mitochondrial dysfunction often results in a massive increase in intracellular ROS, which in turn triggers intrinsic apoptosis [27]. Collectively, our data suggested that K73-03-induced mitochondrial damage largely impairs the cell viability of HepG2 cells. Moreover, the results showed that NAC pretreatment could inhibit the expression of cleaved-caspase9, as shown in Fig. 4D. In conclusion, K73-03 could induce apoptosis and reduce cell proliferation by inducing ROS overproduction and activating the mitochondrial pathway.

### The JAK2/STAT3 and NF-κB/COX-2 signaling pathway were the potential target of K73-03 in HepG2 cells

**Fig. 5:**
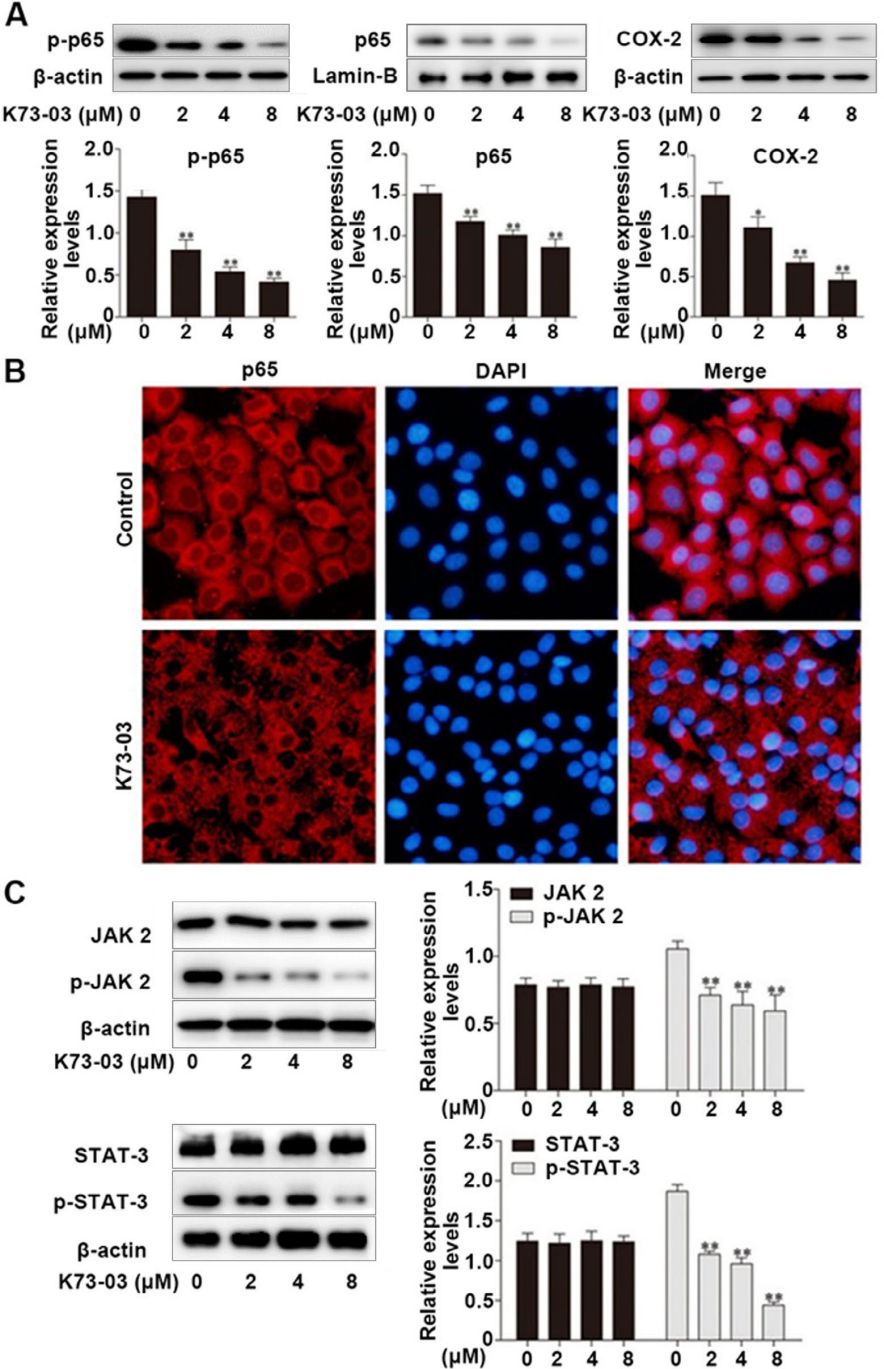
Induction of the p65 transduction in nuclear autophagy and apoptosis by K73-03 in HepG2 cells through activating JAK2/STAT3 signal pathways. (**A**) The expressions of COX-2, p65 in nuclear and p-p65 in cytoplasm in HepG2 cells were analyzed by Western blotting. (**B**) After treatment with K73-03, the subcellular localization of p65 was examined by immunofluorescence staining with inverted fluorescence microscope. (**C**) The expressions of JAK/p-JAK, STAT/p-STAT in HepG2 cells were analyzed by Western blotting. The data were presented as the mean ± SD of three independent experiments. (*p < 0.05, **p < 0.01, significantly different from control group)

Studies have shown that the high expression of cyclooxygenase2 (COX-2) could induce cancer proliferation and migration. NF - κ B is an essential transcription factor regulating COX-2, and the promoter region of the COX-2 gene contains the binding sequence of NF -κB [28]. To determine the change of COX-2 expression after K73-03 treatment on HepG2, the expression of COX-2 was evaluated by western blot. As shown in Fig. 5A, the expression of COX-2 in HepG2 cells was decreased with the increase of the K73-03 treatment. The expression of p-p65 in the cytoplasm and p65 in the nucleus were also evaluated. The results showed that K73-03 inhibited the expression of p-p65 in cytoplasm and p65 in the nucleus in a dose-dependent manner (Fig. 5A). At the same time, immunofluorescence result showed that K73-03 could inhibit p65 nuclear translocation (Fig. 5B).

JAK/STAT could induce cell differentiation, proliferation and apoptosis progress. STAT3 is associated with cancer, cell proliferation, vascularization and immune evasion [29, 30]. As shown in Fig. 5C, western blot analysis showed that K73-03 decreased the phosphorylation of JAK2 and STAT3 after 24 h in a concentration-dependent manner in HepG2 cells. Collectively, these results suggested that K73-03 may not only regress NF - κ B / COX-2 pathway but also inhibit JAK2/ STAT3 in HepG2 cells.

## Discussion

Our previous studies have shown that several new OA derivatives synthesized by us, SZC014, SZC015, and SZC017, induced apoptosis of a variety of human cancer cells [11–18]. In this study, K73-03, our newly synthesized OA derivative, has less toxicity to the L02 cell line (normal liver cell), while having an obvious killing effect on cancer cells, SMMC-7721 and HepG2 cell lines. Moreover, this study found that K73-03 not only induced apoptosis but also autophagy in HepG2 cells.

In the present study, K73-03 induced excessive intracellular ROS generation, ROS is conferred as a kind of signaling molecule to induce autophagic cell death. The potential sources of ROS contain the mitochondrial respiratory chain, xanthine oxidase, NADH/NADPH oxidase and hemoprotein [31–33]. Understanding ROS production will be a valuable tool for us to study new therapeutic targets and ways. Suitable ROS is beneficial while excessive ROS leads to mitochondrial dysfunction, inducing cell apoptosis and causing vacuolization [34]. At the results of our study, we think the formation of vacuoles increased the activity of apoptosis [35, 36]. It has been generally believed that cytotoxicity is one of the reasons for inducing massive macropinocytosis, resulting in catastrophic vacuolization [37]. On the other hand, mitochondria membrane destabilization may be the main reason for the induction of autophagic vacuoles [38]. Besides, ROS assists in promoting cell death during autophagy and apoptosis [39]. The autophagic vacuole formation including many molecular apoptoses is characterized as autophagic degeneration during physiological autophagy in healthy cells [40]. Our results supported the view that apoptosis and autophagy occur simultaneously during vacuolation. This phenomenon is worth further exploring the relationship among them and the underlying mechanism and their logic.

In addition, mitochondria are reported to be involved in their pathological process of them. The mitochondrial function is degraded and autophagy takes place when a few mitochondria are damaged. Moreover, the more mitochondria are damaged, the more cells die resulting from the occurrence of apoptosis and vacuolization [41, 42]. Mitochondrial fission accelerates mitophagy, leading to membrane permeabilization by the autophagic vacuoles, eventually resulting in severe cell death [43, 44]. Our results confirmed that K73-03 caused ROS overgeneration, and subsequently induced the dysfunction of mitochondria, including the decrease of ETC, FCCP, OCR, and ATP, resulting in the occurrence of apoptosis and the formation of vacuoles. It suggested that vacuolization may be involved in the occurrence of apoptosis and autophagy of liver cancer cells [45]. Collectively, these suggested that ROS overgeneration could cause vacuoles. However, the links between them still need to be further investigated.

Apoptosis is a rigorous programmed cell death with a unique biochemical and genetic pathway, which plays a key role in the development and maintenance of normal tissues. Evasion of apoptosis is a significant marker of cancer, and cancer cells can cause apoptosis signal transduction damage, which can promote cancer development and metastasis. At present, inducing apoptosis is the main mechanism of most anticancer drugs. Previous studies have shown that OA could induce apoptosis of cancer cells [7]. Apoptosis is induced in a dose-dependent manner. In the process of apoptosis, Bcl-2 family proteins play a role by interfering with caspase, as the key effector of programmed cell death. It can form ion channels in the biofilms and regulate apoptosis by affecting the permeability of mitochondria. It has been reported that the change in the ratio of Bcl-2 to Bax plays a more important role in the regulation of apoptosis than the change of Bcl-2 family protein level alone. During apoptosis, Bax induces the initiation of the intrinsic apoptotic pathway and forms apoptotic bodies, leading to the release of cytochrome C and the activation of Caspase-9 and Caspase-3, thereby promoting the execution of apoptosis. In this study, K73-03 could increase the ratio of Bax / Bcl-2 and the expression of cleaved-caspase-3 and cleaved-caspase-9 in a dose-dependent manner, suggesting that K73-03 could induce the mitochondrial apoptosis pathway of HepG2 cells.

Autophagy is a highly conserved cell self-protection process that maintains cell homeostasis. After isolation, the cytoplasmic contents are transferred to the lysosome by biomembrane autophagy and degraded. Autophagy ensures the transport of metabolizable substrates to meet the energy requirements during stress, thus supporting cell growth and survival [46, 47]. Although autophagy usually promotes cell survival, over-activation leads to nonapoptotic programmed cell death. At present, the most famous autophagy marker is LC3-II protein [48]. Our results showed that K73-03 induced autophagosome formation accompanied by the enhancement of Beclin1 and ratio of LC3BII/I, suggesting that the phenomenon may be related to one of the mechanisms of anticancer action, resulting from autophagic cell death.

Autophagy and apoptosis are complicated. Under some conditions, autophagy can improve cell survival and avoid apoptosis [49]. In other conditions, autophagy may be consistent with apoptosis or lead to cell death independently when apoptosis fails. At present, most cancer studies involve both regards to different pathways. For example, the p53 signaling pathway plays a crucial role in restraining the growth of mutated cancer cells by promoting apoptosis, autophagy, and other stress responses [34]. Moreover, the STAT3 signaling pathway has the ability to influence apoptosis and autophagy, leading to the destruction of cancer cells. When STAT3 is activated, it can alter the levels of Bax and Bcl-2, resulting in a decrease in apoptosis. Additionally, the activation of STAT3 also impacts molecules associated with autophagy. Deficiencies in Beclin-1 and ATG7 make individuals highly susceptible to septic shock induced by LPS, partially due to the activation of STAT3 signaling [50]. Collectively, our study has found the coexisting phenomenon of apoptosis and autophagy-induced cell death, which present apoptotic cell death by inhibiting JAK2/STAT3 pathway and NF-κB/P65 pathway respectively, and autophagic cell death by observing the overproduction of Beclin1, IC3BII/I and autophagosomes. However, the in-depth mechanisms of complex interactions between apoptosis and autophagy-induced cell death by K73-03 treatment are still unclear, which is also one of our interesting points and goals that we want to accomplish in the future.

Mitochondria release a variety of pro-apoptotic factors into the cytoplasm, which plays an important role in complex apoptosis. Therefore, mitochondria-mediated apoptosis is a very important internal apoptosis pathway. Mitochondrial integrity is controlled by various members of the Bcl-2 superfamily [51]. Increasing the permeability of mitochondrial membrane and reducing its potential induces caspase activator release rapidly [52]. In this study, we examined the changes of ∆Ψm in HepG2 treated with K73-03. The results showed that K73-03 could decrease the ∆Ψm in a dose-dependent manner. At the same time, we also detected the effect of K73-03 on mitochondrial respiratory function of HepG2 cells. The results showed that the respiratory rate, including ETC, FCCP, OCR, and ATP, in HepG2 cells was significantly decreased by K73-03, suggesting that mitochondrial dysfunction occurred in HepG2 cells. When the mitochondrial function is not completed, a large number of ROS will be generated, which will activate the mitochondrial apoptosis pathway. At present, many chemotherapy drugs play an anti-cancer role by inducing excessive ROS production, and the intrinsic apoptosis pathway is particularly sensitive to ROS [53, 54]. In this study, K73-03 could induce ROS production in a dose-dependent manner, suggesting that ROS may also play an important role in the apoptosis of HepG2 cells induced by K73-03.

Currently, it is still unknown how JAK/STAT signaling affects ROS overproduction, including tyrosine kinase, related receptors, JAK and transcription factor STAT. However, there is some study evidence that STAT is activated in ∆Ψm, while JAK kinase seems to be involved in it [55]. Moreover, both p65 and p-p65 are associated with cancer genesis, cell proliferation and angiogenesis, which is activated in liver cancer tissue and significantly expressed in liver cancer cells with poor prognosis [56]. Taken together, mitochondria mediate apoptosis, cell cycle arrest and inhibition of JAK/STAT signaling pathway in liver cancer cells. And JAK/STAT is ultimately targeted to the inner mitochondrial membrane. It was reported that JAK/STAT signaling pathway may play a vital role in hematological malignancies, prostate cancer and so on [57]. Therefore, the development of inhibitors targeting on JAK/STAT signaling pathway is of great significance in the treatment of various cancers in the future, which is closely related to ROS overgeneration. Our study showed that K73-03 could decrease ROS overgeneration, suggesting JAK/STAT may be one of the pathways involved in the induction of HepG2 cells apoptosis, autophagy or vacuolization. Therefore, we speculated that K73-03 may participate in the process of inactivation of JAK2/STAT3 pathway. Our results authenticated our above assumption, showing the decrease of expressions of p-JAK2 and p-STAT3 respectively. However, it remains uncertain how K73-03 acts on JAK2/STAT3 pathway, directly or indirectly? Hence, it is necessary to further study the relationship between JAK2/STAT3 pathway and inhibition of K73-03 on liver cancer cells.

COX-2 is an enzyme involved in tumorigenesis and development, which plays an important role in regulating cancer cell growth, migration, invasion and inflammatory reaction. COX-2 is overexpressed in malignant tumor tissues [58]. Studies have shown that COX-2 inhibitors (including NSAIDs) prevent a variety of tumors [59–61]. NF - κ B is an important transcription factor in regulating COX-2. The promoter region of COX-2 gene contains the binding sequence of NF - κ B [62]. Studies have confirmed that activation of NF - κ B signal induces the expression of COX-2, meaning that the anticancer effect of drugs may be accompanied by the down-regulation of COX-2 expression. Inhibition of NF - κ B pathway leads to cancer cells being more sensitive to apoptosis induced by anticancer drugs. NF- κ B is expressed in liver epithelial cells, which regulates the proliferation and survival of hepatocytes. Some studies have shown that the activation of NF - κ B has an effect on all aspects of liver cancer progression [63–65]. In addition, RelA / p65 is closely related to inflammation, cell proliferation and cancer in NF - κ B family. Activation of NF - κ B / p65 regulates many biological functions, and the down-regulation of NF - κ B pathway reduces the viability of cancer cells [66, 67]. In this study, K73-03 could reduce the expression of COX-2 in a dose-dependent manner. Besides, K73-03 could reduce the content of p65 in nucleus and p-p65 in cytoplasm, and inhibit p65 nuclear translocation, suggesting that K73-03 may down-regulate the NF - κ B / P65 pathway and induce the apoptosis of HepG2 cells. This study revealed that K73-03 could cause mitochondrial dysfunction including the decrease of ETC, FCCP, OCR, ATP, producing excessive ROS in HepG2 cells. We have demonstrated that the vacuoles are closely associated with inducing cell apoptosis and autophagy, making NF- ĸB/P65 translocating into the nucleus and the upregulation of COX-2 through the inhibition of JAK2 / STAT3 pathway and NF - κ B / P65 pathway.

## Conclusion

**Fig. 6:**
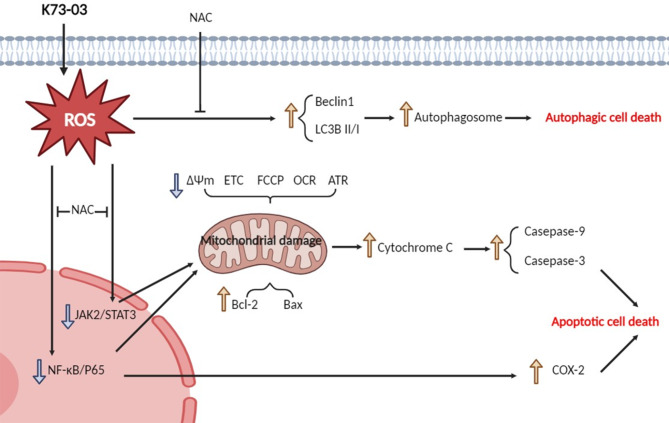
Schematic diagram showing mechanism of K73-03 on the induction of cell autophagy and apoptosis in hepatocellular carcinoma. K73-03, a derivative of oleanolic acid, can increase the generation of ROS, resulting in not only inducing autophagosome accompany with the enhancement of Beclin1 and ratio of LC3BII/I, ultimately leading to autophagic cell death, but also stimulating JAK2/STAT3 and NF- ĸB/P65 signals. Both of them can be alleviated by NAC (an active oxygen scavenger). Subsequently, one side, mitochondria was damaged, manifesting by the increase of Bcl-2, Bax and the decrease of ∆Ψm, ETC, FCCP, OCR, ATP, causing the release of Cytochrome C, the activation of Casepase-9, Casepase-3, eventually the occurrence of apoptotic cell death. On the other side, NF- ĸB/P65 translocated into nucleus after the activation of these two signals, bringing about the upregulation of COX-2, further aggravating apoptotic cell death

In summary, K73-03 could increase the generation of ROS, resulting in not only inducing autophagosome accompanied by the enhancement of Beclin1 and the ratio of LC3BII/I, ultimately leading to autophagic cell death, but also stimulating JAK2/STAT3 and NF- ĸB/P65 signals. Both could be alleviated by NAC. Subsequently, for these two signals, on one side, mitochondria were damaged, manifesting by the increase of Bcl-2, Bax and the decrease of ETC, FCCP, OCR, ATP, causing the release of Cytochrome C, the activation of Casepase-9, Casepase-3, eventually the occurrence of apoptotic cell death. On the other side, NF- ĸB/P65 translocates into the nucleus after the activation of these two signals, bringing about the upregulation of COX2, further aggravating apoptotic cell death (Fig. 6). This study provides a preliminary basis for further cancer treatment of hepatocellular carcinoma. However, follow-up studies of K73-03 and its mechanism involved still need to be explored.

### Electronic supplementary material

Below is the link to the electronic supplementary material.

Supplementary Material 1

## Data Availability

The authors declare that all data supporting the findings of this study are available within the article.

## Abbreviations

∆Ψm: Mitochondrial membrane potential
COX2: Cyclooxygenase2
ETC: Mitochondrial electron transport chain
FCCP: Carbonyl cyanide 4- (trifluoromethoxy)phenylhydrazone: a kind of mitochondrial uncoupling agent
JAK2: Janus kinase 2
L02: HL-7702 cell line
MRC: Maximum respiratory rate
NAC: N-acetyl-L-cysteine
OA: Oleanolic acid
OCR: Oxygen Consumption Rate
PI: Propidium iodide
ROS: reactive oxygen species
STAT3: Signal Transducer and Activator of Transcription signal
TBS: Tris Buffered Saline
TCM: Traditional Chinese medicine
TEM: Transmission electron microscope
