# Supplementary figures and images for: Mechanism study of oleanolic acid derivative, K73-03, inducing cell apoptosis in hepatocellular carcinoma
Source: Cancer Cell Int. 2024 Jan 7;24:17. doi: 10.1186/s12935-023-03119-x (PMC10771654; doi:10.1186/s12935-023-03119-x)

**Figure 2D-1**


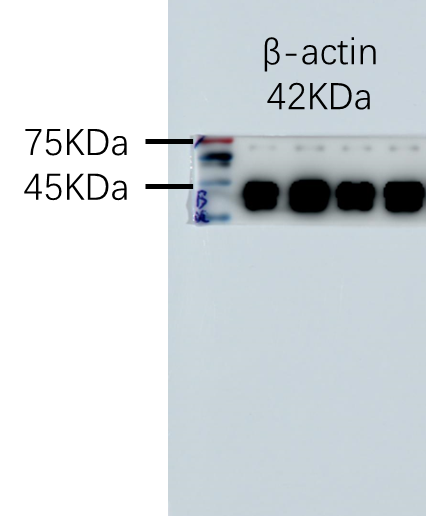

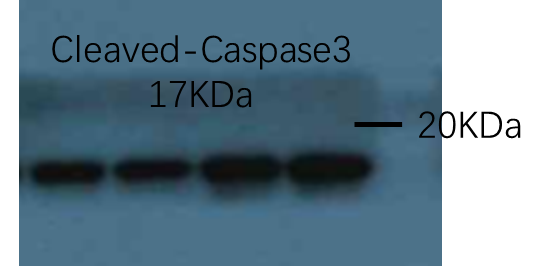

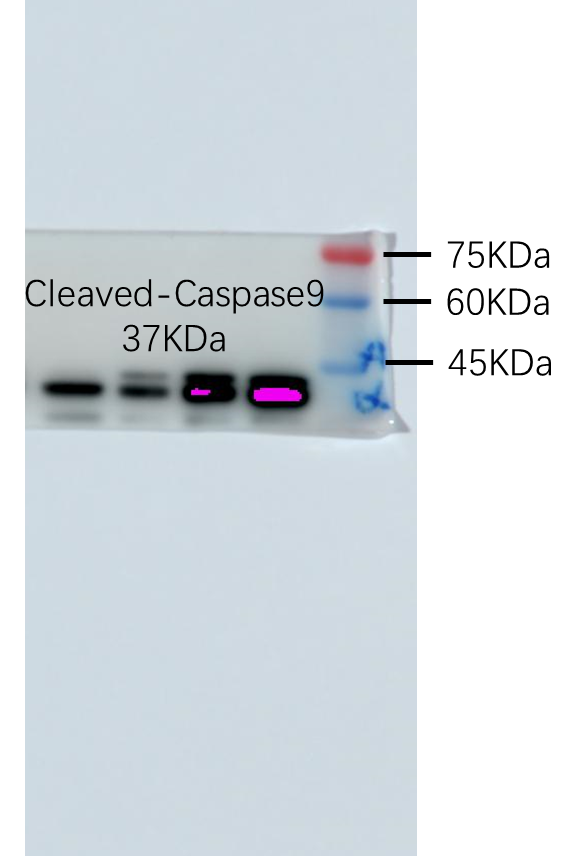


**Figure 2D-2**


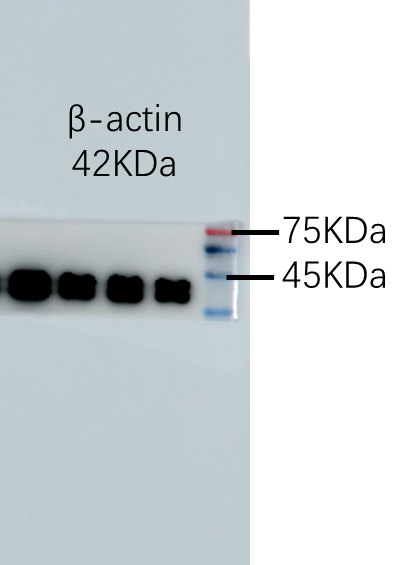

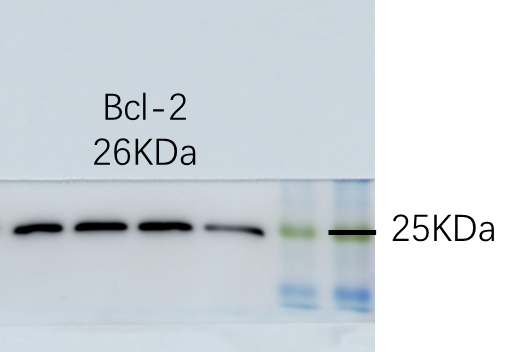

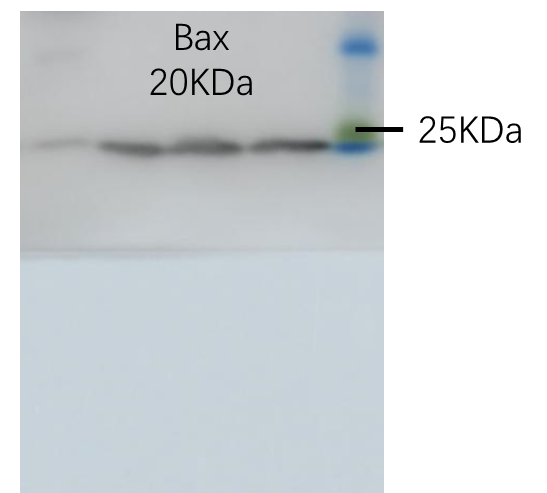


**Figure3B-1**


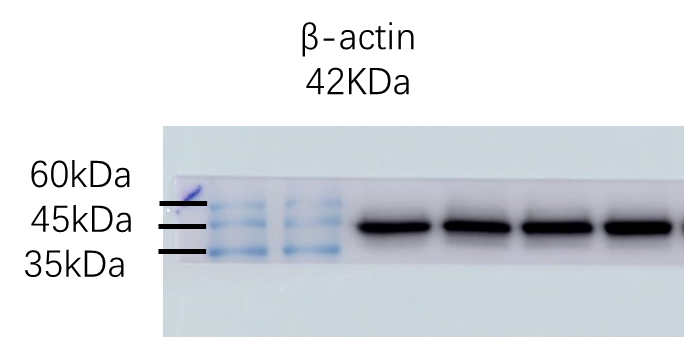

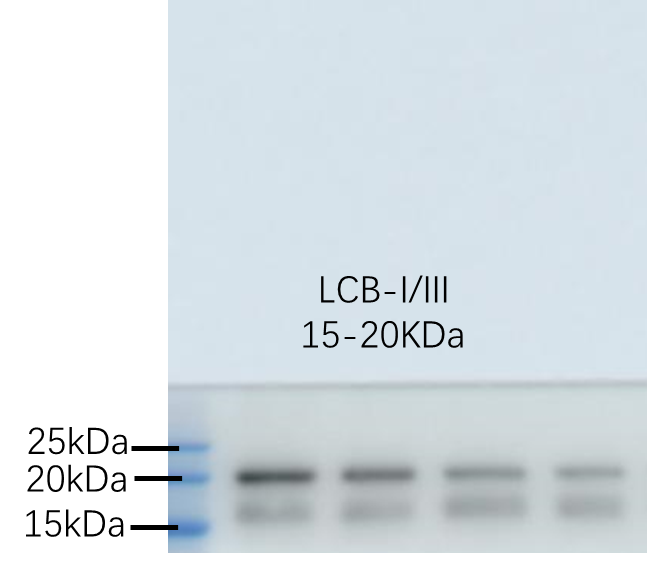


**Figure3B-2**


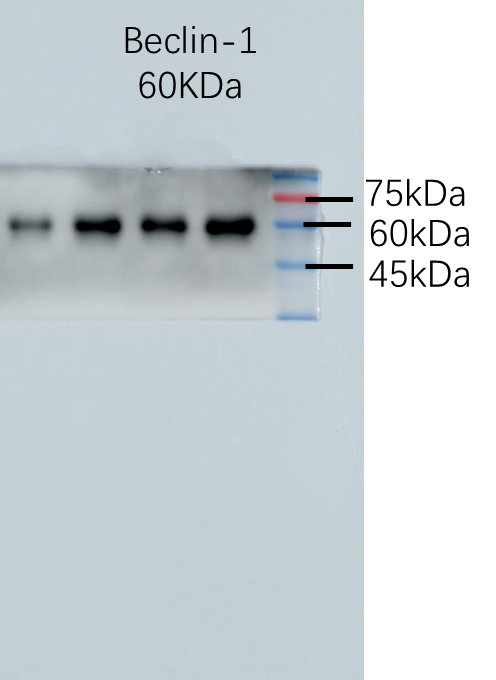


**
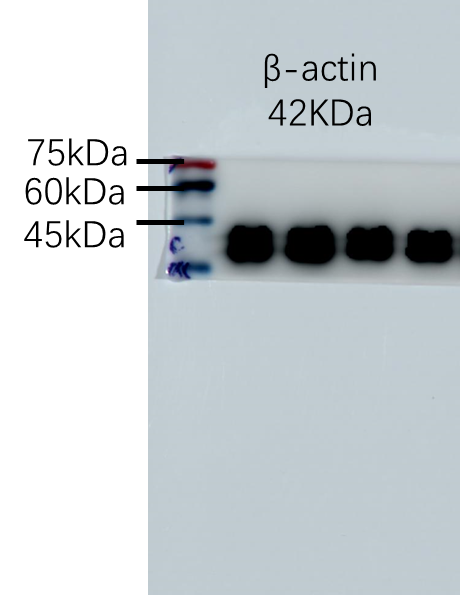
**

**Figure4D**

**
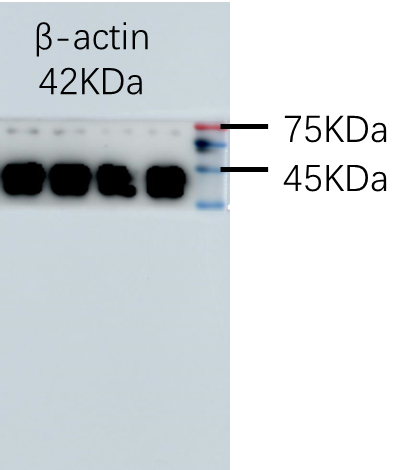

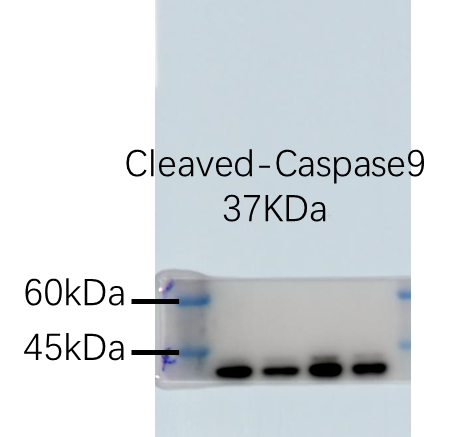
**

**Figure5A-1**

**
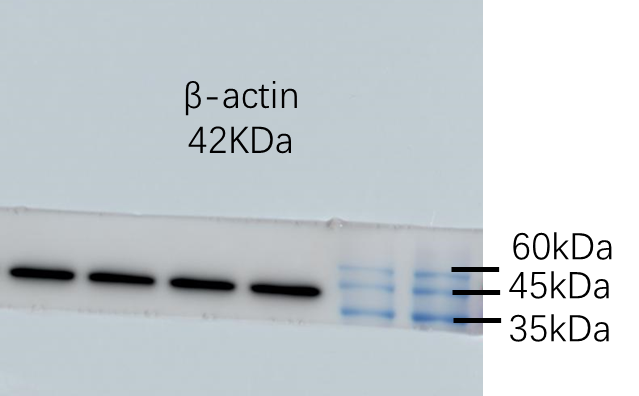

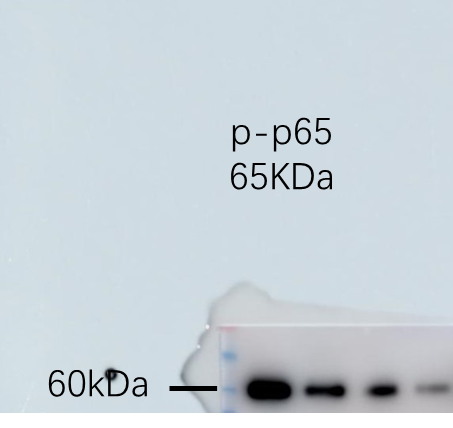
**

**Figure5A-2**

**
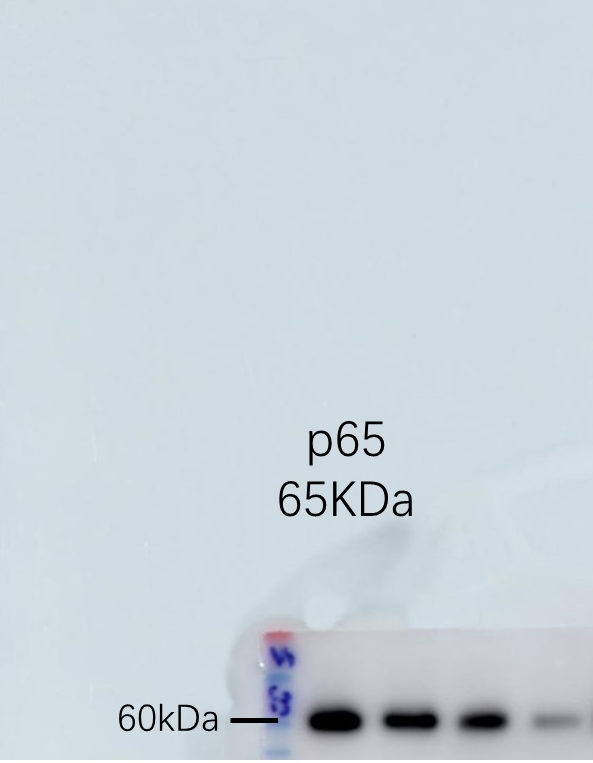
**


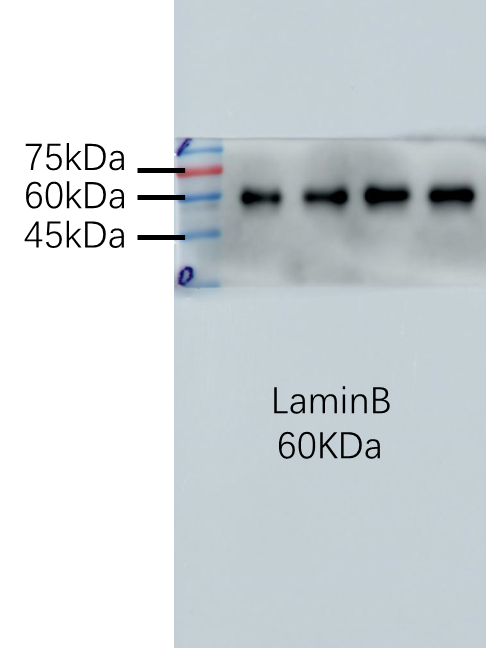


**Figure5A-3**


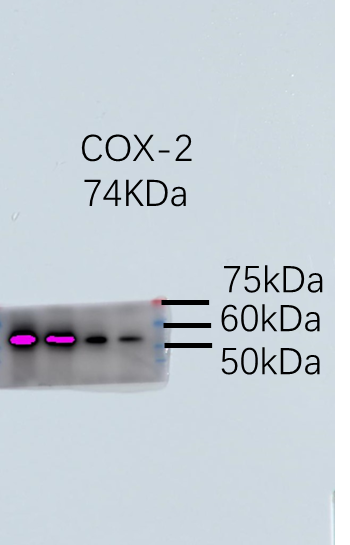

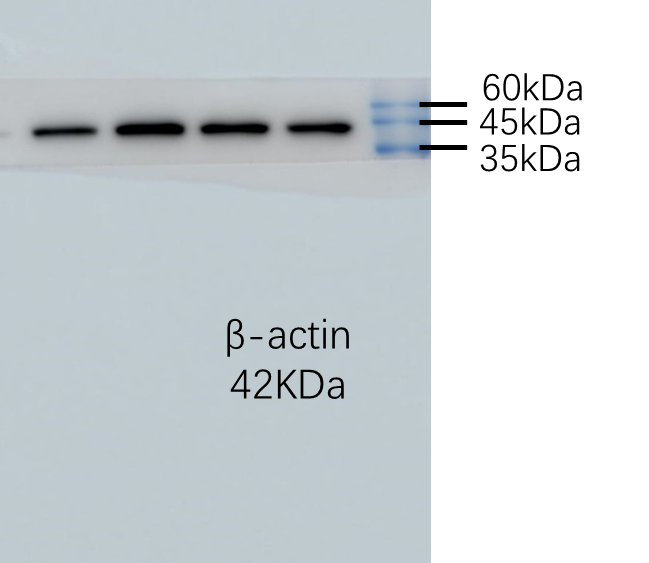


**Figure5C-1**


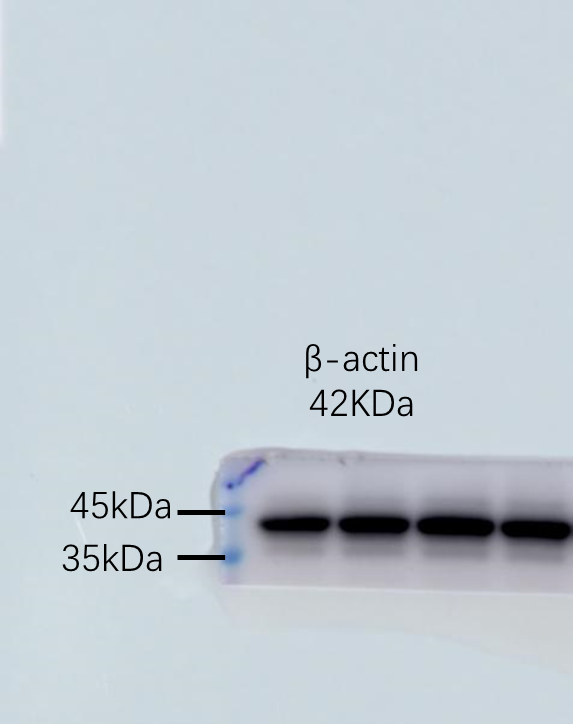

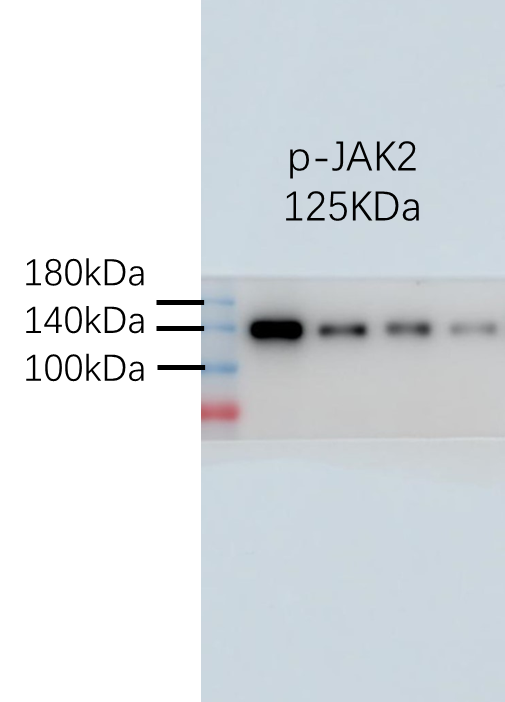

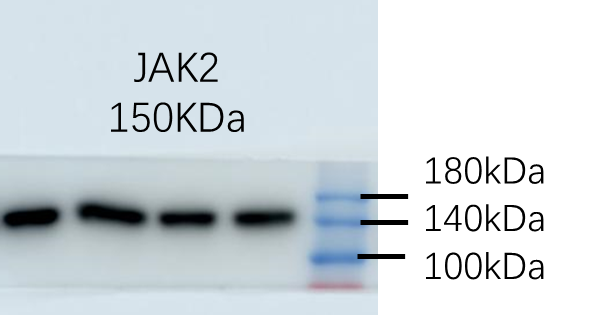


**Figure5C-2**


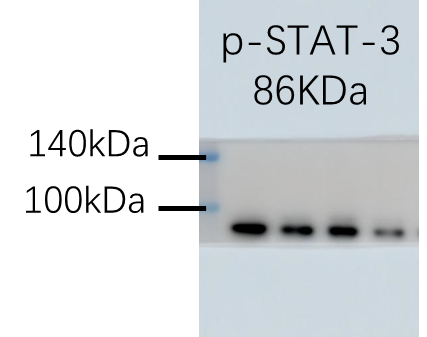
**
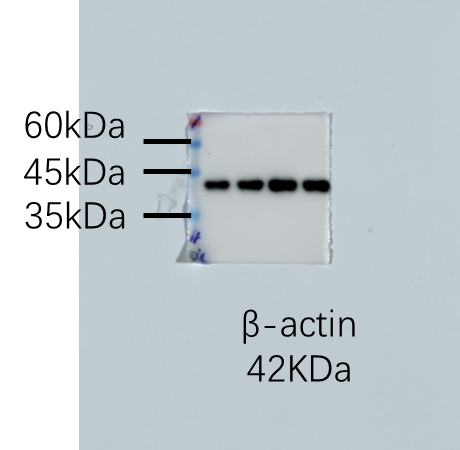
**
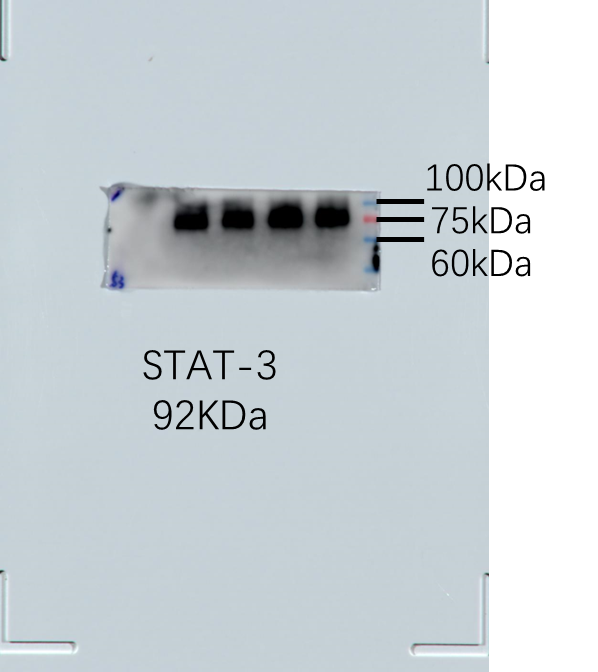

Supplement: Supplementary file 1 — Supplementary Material 1 [file 12935_2023_3119_MOESM1_ESM.docx]
